# Impact of Work Goals on Quiet Quitting Among Chinese Primary Health Professionals Based on Goal Setting Theory: A Cross-Sectional Survey

**DOI:** 10.3390/healthcare13212739

**Published:** 2025-10-29

**Authors:** Jinwen Hu, Dongdong Zou, Qianqian Xu, Yuanyang Wu, Si Fan, Yanting Wang, Xinping Zhang

**Affiliations:** School of Medicine and Health Management, Tongji Medical College, Huazhong University of Science and Technology, Wuhan 430030, China; m202375931@hust.edu.cn (J.H.); m202375932@hust.edu.cn (D.Z.); d202381924@hust.edu.cn (Q.X.); d202281850@hust.edu.cn (Y.W.); fans_hust@163.com (S.F.); w2279234150@163.com (Y.W.)

**Keywords:** goal setting theory, work goals, quiet quitting, primary health professionals, workforce motivation

## Abstract

**Background**: Goal setting has always been a crucial management factor for workforce motivation and is quite complex due to multiple goal characteristics. Considering that the emergence of Quiet Quitting (QQ) has inflicted harm on employees’ mental well-being in the healthcare field, urgent attention needs to be paid to the impact of goal setting on QQ. This study aimed to assess the current state of work goal setting and QQ among primary health professionals and to explore the effect of goal characteristics on QQ. **Methods**: A cross-sectional study was performed among 520 primary health professionals from 11 primary health centers. The Modified Goal Setting Scale and Quiet Quitting Scale were utilized to measure goal characteristics and QQ. Descriptive analysis, cluster analysis, and multiple regression were used for statistical analysis. **Results**: The mean score of QQ was 2.12. The eight goal characteristics were clustered into five categories. Among them, two categories demonstrated significant negative effects on QQ: Goal Specificity and Identity (Category 1; β = −0.096, *p* < 0.05) and Goal Fulfillment and Organizational Support (Category 2; β = −0.466, *p* < 0.001). Conversely, three categories showed significant positive effects: Goal Difficulty (Category 3; β = 0.112, *p* < 0.05), Goal Attainability (Category 4; β = 0.142, *p* < 0.01), and Goal Conflict (Category 5; β = 0.185, *p* < 0.001). **Conclusions**: The phenomenon of QQ requires attention among Chinese primary health professionals. Setting work goals scientifically may prove to be beneficial in curbing its spread. From a practical perspective, goal setting should be specific, moderately challenging, yet attainable, recognized and accepted by employees, and strongly supported by the organization. This approach is valuable for reducing QQ and fostering supportive work environments in primary healthcare. It should be noted, however, that while this study identifies significant associations, its cross-sectional design precludes causal inference, and the findings are context-specific to Chinese primary healthcare institutions.

## 1. Introduction

Goal setting has been widely applied in the global health field for workforce motivation [1]. From the Alma-Ata Declaration of 1978, which put forward the goal of “primary health care for all by the year 2000”, to the WHO’s goal of “achieving universal health coverage by 2030”, setting health goals is undoubtedly integral to health professionals’ motivation and human health. But goal setting is quite complex, and it must consider multiple goal characteristics, such as goal specificity, goal difficulty, goal commitment, and goal attainability [2,3,4,5]. With questionnaires and scales on relevant characteristics [4,6,7,8,9,10], goal setting has been widely researched in sport psychology [11,12], cognitive psychology, and educational psychology [13,14,15,16], which mainly focus on goal specificity and goal difficulty [17]. Therefore, it is both necessary and innovative to conduct a comprehensive assessment of goal characteristics in order to provide evidence for improving goal setting among primary health professionals—an endeavor that is valuable for motivating the workforce.

“Quiet Quitting” (QQ), sparked by TikTok (version 24.4.3) videos, has become one of the most publicized workplace-related topics on social media in 2022. QQ describes a situation where employees only undertake tasks explicitly outlined in their job descriptions, and are reluctant to engage in additional responsibilities beyond what is assigned [18]. Essentially, QQ is a kind of spiritual turnover behavior with concealment [19]. It does not constitute an actual resignation, but rather represents an employee’s conscious decision to reduce their performance and productivity levels while choosing to remain within the organization [20,21]. This phenomenon differs from the extensively researched concept of job burnout, which is typically characterized by emotional exhaustion, depersonalization, and a diminished sense of personal accomplishment [22]. In contrast, QQ should be understood as a covert strategy or defensive behavior in response to work-related stress, constituting a modern workplace psychological phenomenon that remains insufficiently understood [23]. Gallup’s State of the Global Workplace 2023 survey shows that quiet quitters are prevalent in modern organizations, with at least 59% of workers being QQ [24]. All of these findings underscore a deep, prolonged, and widespread workforce dissatisfaction that cannot be ignored any longer and must be addressed [25].

Some scholars have done research on QQ. Harter believed QQ is related to a growing disconnect between employees and their employers, including a lack of connection to organizational goals [25]. This disconnect often reflects employees’ negative perceptions of the goal characteristics set by the organization, such as their reasonableness and feasibility. Based on goal setting theory, as specific work goals help employees focus on essential tasks and minimize resource wastage, goals have motivational effects [2]. Challenging goals motivate employees to work harder and adopt more efficient strategies [26]. However, when employees perceive their occupational goals as unattainable, it may cause disappointment and frustration, which ultimately affects job commitment and job performance [27] and becomes a key risk factor for QQ. Understanding the organization’s goals strengthens employees’ alignment with its values [28]. Furthermore, when employees actively participate in determining their work goals, they will also create a stronger feeling of ownership towards their roles, leading to increased job satisfaction and performance outcomes [29]. Conversely, if employees are excluded from the goal-setting process, they may resort to QQ as a response to feeling undervalued and losing a sense of control. Although goal setting theory has been widely applied in organizational management and some studies have linked employees’ work goals to burnout and QQ [30,31,32], few studies have investigated the association between multidimensional goal characteristics and QQ systematically. This is especially understudied in resource-constrained primary healthcare settings.

Therefore, it is necessary to explore the impact of goal characteristics on QQ among primary health professionals, especially in remote areas, where the lack of human resources and extrinsic motivators is particularly pronounced [33]. The expected results can provide evidence-based suggestions for optimizing goal setting and reducing QQ in primary health organizations.

## 2. Materials and Methods

### 2.1. Study Design and Sampling

A cross-sectional, face-to-face survey was conducted during January 2024 across all 11 primary health centers in Yingshan County, Hubei, China, which collectively employed approximately 688 health professionals. This region is a model county recognized for intensive and effective healthcare management reforms that emphasize the motivational role of work goals. Participants were health professionals who were on duty, had the time and willingness, and were required to complete the questionnaire. Professionals who had worked for less than half of their working years were excluded.

Data were collected through face-to-face interviews by researchers following the principle of full sampling. First, researchers explained the purpose and significance of the study to eligible professionals using standardized instructions and obtained their written informed consent. Participants were then invited to complete a structured paper questionnaire independently in a conference room, typically taking 15–20 min. Upon submission, 2 researchers carefully reviewed each questionnaire to ensure completeness and accuracy. Invalid questionnaires were excluded according to the following criteria: (1) unreasonable answers, such as implausibly long clinical work years; (2) short response time (less than 10 min), which was below the minimum time required for reliable completion as established by our research group; and (3) inconsistent responses to trap items—pairs of questions with similar meaning but different wording that were embedded within the questionnaire to detect random or inattentive responding. Finally, a total of approximately 540 questionnaires were distributed, with 528 completed and returned, yielding an overall response rate of 97.78%. After excluding invalid samples, 520 valid questionnaires were retained, resulting in a final valid response rate of 98.48%.

### 2.2. Measurements

To clarify the perspective adopted in this study, in goal setting, primary health centers set work goals through task assignments, performance appraisal, and management systems. Professionals’ responses to the questionnaire are based on their personal perceptions and understanding of the goals. The questionnaire was developed with reference to the Goal Setting Scale constructed by Yukl et al. [6,8,34], and the Quiet Quitting Scale by Galanis et al. [35,36,37]. The questionnaire consisted of three sections: goal characteristics, QQ, and demographics of health professionals.

Specifically, goal characteristics were measured through 36 items across 8 dimensions. Each dimension, along with its source and sample items, is presented below: (1) goal specificity with 7 items (for example, “The organization for which I work has clearly defined goals”, “I have specific and clear goals to aim for on my job”) [3,38]; (2) goal difficulty with 4 items (for example, “I find it difficult to achieve the work goals”) [6,8,39]; (3) goal identify with 4 items (for example, “I fully identify myself with the work goals”) [34,40,41]; (4) goal acceptance with 3 items (for example, “I was committed to attaining my work goals”) [6,8]; (5) goal commitment with 6 items (for example, “I am willing to put forth a great deal of effort beyond what I’d normally do to achieve work goals”) [7,42]; (6) goal attainability with 4 items (for example, “I am confident of my success in pursuing my work goals”) [34,43]; (7) goal conflict with 3 items (for example, “My work goals lead me to take excessive risks”) [3]; (8) organization facilitation of goal achievement with 5 items (for example, “This organization provides sufficient resources to make goal setting work”) [3].

For QQ, 6 items (for example, “I do the basic or minimum amount of work without going above and beyond”, “If a colleague can do some of my work, then I let him/her do it”) were rated on a five-point Likert scale as follows: (1) strongly disagree, (2) disagree, (3) neither disagree or agree, (4) agree and (5) strongly agree. Three items were reverse-scored (i.e., “I give my best at work”, “I find motivation in my job”, “I feel inspired when I work”). Higher values indicated higher levels of QQ. In this study, the internal reliability was considered acceptable, with the Cronbach’s alpha coefficients of 8 goal characteristics and QQ being 0.948, 0.714, 0.927, 0.851, 0.800, 0.753, 0.842, 0.939, 0.863, respectively.

### 2.3. Statistical Analysis

Data were checked, coded, and entered into the Sojump (version 2.2.78) platform. Then, the data were exported to SPSS 22.0 for further analysis. Descriptive statistics for continuous variables (with normality assessed by the Shapiro–Wilk test) are presented as means and SD, while categorical variables are summarized as frequencies and percentages. R-type cluster analysis was applied to cluster the goal characteristics, in which Euclidean distances were used to determine the degree of closeness between the variables. Multiple regression analysis was applied to model the factors influencing QQ, with a test level of α = 0.05.

## 3. Results

### 3.1. Distribution of Goal Characteristics and QQ

The mean scores for goal specificity, goal difficulty, goal identity, goal acceptance, goal commitment, goal attainability, goal conflict, and organization facilitation of goal achievement ranged from 2.66 to 4.30. The mean score for QQ is 2.12. Other specifics are shown in Table 1.

### 3.2. Univariate Analysis of Characteristics on QQ

Among 520 participants, 189 were male and 331 were female; the numbers of participants from clinical, clinical examination, hospital administration, public health, and other departments were 179, 71, 78, 118, and 74, respectively. Other specifics are shown in Table 2.

The one-way analysis of variance showed that the factors significantly associated with QQ included gender, health status, job position, length of service, and type of employment. Specifically, male participants had higher levels of QQ than female participants; “healthy” professionals had the lowest levels of QQ; professionals with 30–40 years of service had the highest levels of QQ; this group also had the highest mean scores in six of the nine QQ sub-items, such as “finding motivation at work” (2.00), “being inspired at work” (2.09) and “doing only basic work” (2.53); QQ levels were relatively low in administrative and public health departments, while the highest levels were observed in clinical examination departments, especially in the clinical departments.

### 3.3. Cluster Analysis of Goal Characteristics

**Figure 1:**
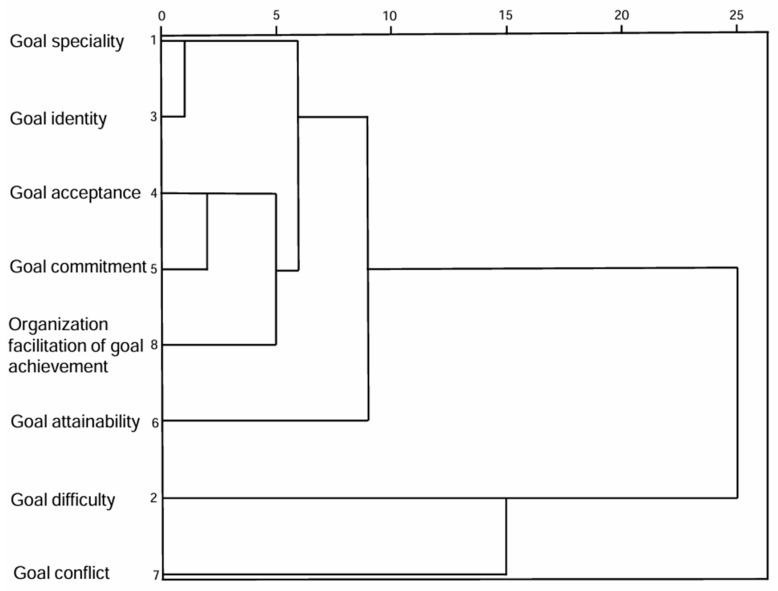
Dendrogram of r-clustering results.

This study explored the similarities and differences among the goal characteristics by clustering the variables into 2 to 5 categories, respectively. Figure 1 shows that when the variables were clustered into 3–5 categories, respectively, goal difficulty and goal conflict were all significantly different from other variables, each being classified as a separate category due to relatively independent sources and causes.

Based on the clustering results, goal characteristics were divided into 5 categories: goal specificity and goal identity were classified as Category 1, and named “Goal Specificity and Identity”; goal acceptance, goal commitment, and organization facilitation of goal achievement were classified as Category 2, and named “Goal Fulfillment and Organizational Support”; Goal Difficulty, Goal Conflict, and Goal Attainability were each classified as a separate category. The mean scores for goal categories are shown in Table 3.

### 3.4. Regression Analysis of Goal Categories on QQ

To test the relationship between goal categories and QQ, we performed a correlation analysis. As shown in Table 4, all goal categories were correlated with QQ. The analysis showed that goal category levels were closely related to QQ.

We then performed hierarchical regression to examine the effects of the five goal categories on QQ. The first step was to explore the influence of demographic variables. The second step was to test the five goal categories. As shown in Table 5, all goal categories had significant effects on QQ. When controlling for demographic variables, Category 1—Goal Specificity and Identity (*p* < 0.05), Category 2—Goal Fulfillment and Organizational Support (*p* < 0.001) had significant negative effects on QQ, and Category 3—Goal Difficulty (*p* < 0.05), Category 4—Goal Attainability (*p* < 0.001), and Category 5—Goal Conflict (*p* < 0.01) had significant positive effects on QQ. For controlled variables, health status and job position had significant effects on QQ, while other characteristics did not have a significant effect on QQ.

## 4. Discussion

This study yielded three main findings. First, QQ among health professionals was observed at a moderately high level. Second, goal characteristics were grouped into five core categories. Third, Category 1—Goal Specificity and Identity, Category 2—Goal Fulfillment and Organizational Support all had negative effects on QQ, whereas Category 3—Goal Difficulty, Category 4—Goal Conflict, and Category 5—Goal Attainability had positive effects on QQ.

### 4.1. QQ and Goal Categories

First, this study found that QQ among primary health professionals was at a moderately high level. This finding coincides with related studies, as Xu et al. point out that 47.6% of health professionals in rural areas are experiencing moderate burnout, and 3.3% of participants are in a state of severe burnout [44]. In Li et al.’s study, the job satisfaction, burnout, and willingness to leave of health professionals were 79.99%, 18.69% and 26.04%, respectively [45]. Health professionals, compared to the general population, are confronted with more stress and workload and are more vulnerable to mental disorders [46]. Therefore, it is important to pay attention to QQ among Chinese primary health professionals and to identify the associated factors that could potentially be addressed to lower the risk of QQ.

Second, through the cluster analysis of multiple goal characteristics, we found they could be summarized into five core categories, namely Goal Specificity and Identity, Goal Fulfillment and Organizational Support, Goal Difficulty, Goal Attainability, and Goal Conflict. The results of this study provide unique insights and effective methods for scientifically setting and quantitatively evaluating goals, especially for primary healthcare organizations. The category division helps to analyze the essential characteristics of each task deeply, to set more reasonable and effective work goals, and to guide health professionals to examine their own work goals from a multivariate perspective, ultimately reducing the occurrence of QQ among professionals.

### 4.2. Goal Characteristics Impacting QQ

The study found that Goal Specificity and Identity, Goal Fulfillment and Organizational Support could reduce the level of QQ, while Goal Difficulty, Goal Conflict, and Goal Attainability could increase the probability of QQ.

#### 4.2.1. Positive Impact of Goal Specificity and Identity on QQ

This study concluded that Goal Specificity and Identity could significantly reduce the level of professionals’ QQ. This finding aligns with the research from Swedish primary healthcare centers, which highlights that clear direction of goals and consistency between individual and organizational goals help motivate professionals and provide space for quality improvement work [47]. This also quantitatively confirms Mento et al.’s view [48] from another perspective, that specific goals can motivate people to pursue higher goals, and that goal identity is also closely related to goal motivation [49]. Specifically, communicating a clear vision of the future is an essential tool for motivating employees [50]. And clear work goals give professionals a clear standard to measure their efforts, which is conducive to maintaining enduring work enthusiasm and concentration, and reduces the likelihood of QQ [48]. Individuals without specific goals, on the other hand, may not only lose motivation to pursue higher goals but also tend to overestimate their performance in the face of negative feedback [51]. At the same time, professionals who identify with their work goals are more likely to be intrinsically motivated. They may not only be satisfied with completing basic work tasks, but also willing to devote more energy and time to their work, thus reducing the probability of QQ. When goals are both specific and recognized by professionals, it shows that professionals not only know exactly what to do, but also understand why they need to do it. This helps to promote professionals to be more proactive in their work. This also suggests that when setting goals, managers should take into account the specificity of the goals and the emotional identification of the professionals, so as to reduce the possibility of QQ.

#### 4.2.2. Positive Impact of Goal Fulfillment and Organizational Support on QQ

We found that Goal Fulfillment and Organizational Support could reduce the level of QQ, which provides an evidence-based basis for understanding the relationship between the two. The result is also consistent with the conclusions of the academic community that goal acceptance motivates individuals to put in enough effort to achieve their goals [52,53,54]. Those who possess a commitment to their goals are more likely to take action towards them [55]. While work resources provided by the organization also contribute to achieving work goals, promoting personal development, and reducing psychological strain. Specifically, when professionals accept organizational goals, they internalize them as personal work motivation, thereby fostering engagement and reducing the likelihood of QQ. Furthermore, participation in goal setting is considered an effective strategy for coping with low acceptance [56], as participatory decision-making enhances mutual understanding among organizational professionals and increases individuals’ sense of control, thereby reducing anxiety. Goal commitment is the extent to which individuals perceive goals to be important [57]. Individuals with high goal commitment will devote more energy and resources to goal striving and are more likely to consistently persevere in the struggle [55,58]. The organization provides work resources for professionals in order to achieve organizational goals. Professionals who perceive organizational support will also feel responsible for helping the organization reach its goals and expect to put more effort into the organization in order to bring more rewards [59]. This is further supported by a Turkish study, which confirms that organisational support plays a significant role in reducing quiet quitting behaviour among nurses [60]. Goal acceptance reflects the degree of understanding and acceptance of the professionals’ organizational goals, while goal commitment further extends to the professionals’ determination and loyalty to the goals, and finally, organizational facilitation of goal achievement reflects the all-around support for goal achievement at the organizational level. This also provides important insights for managers: in the practice of goal management, these three dimensions should be emphasized and coordinated to maximize the mobilization of employee potential and reduce the phenomenon of QQ.

#### 4.2.3. Impact of Goal Difficulty and Goal Attainability on QQ

Effective goal setting lies in the balance of Goal Difficulty and attainability [2]. We provide an evidence-based rationale for this association through our empirical study. The results of the study partially differ from previous findings. Previous studies have noted that Goal Difficulty promotes effort, strategy, and sustained effort over time [26]. However, some studies have found that the hypothesis that “difficult goals are more likely than easy goals to lead to higher activity levels” was not confirmed, and that only moderately difficult goals led to higher activity levels [61]. On the one hand, the pursuit of high-performance goals can potentially lead to unethical behavior [62]. On the other hand, professionals facing such pressures are also more likely to enter a state of self-attrition [63]. In the results of this study, both Goal Difficulty and Goal Attainability had significant positive effects on QQ, indicating that goal setting should seek a moderate difficulty range that is both challenging and accounts for professionals’ available resources and actual abilities.

Notably, the positive association between Goal Attainability and QQ runs contrary to intuition. This counterintuitive finding can be effectively interpreted through both the Job Demands-Resources (JD-R) theory and the context of Chinese workplace culture. From the JD-R perspective [64,65], highly attainable goals constitute a form of low-challenge job demand. This type of demand fails to stimulate employees’ interest and focus, potentially triggering boredom and detachment. Simultaneously, such goals lack the capacity to provide key psychological resources—such as a profound sense of accomplishment, competence feedback, or skill development—essential for sustaining long-term motivation. This combination of high demands and low resources readily leads to emotional exhaustion. Complementarily, within China’s prevalent “culture of overtime,” where intensive work symbolizes dedication, easily achievable goals may be perceived as signaling lower organizational expectations and a lack of recognition as core talent. This sense of being undervalued can foster QQ as a strategic form of disengagement through which employees express dissatisfaction or reassert their professional worth.

In summary, while challenging goals can motivate achievement-oriented professionals by fulfilling their growth needs, they may provoke frustration and self-doubt—and ultimately QQ—among those with lower confidence or still in the learning stage. These findings collectively suggest that effective goal setting must account for both the structural characteristics of the goals and the psychological predispositions of the professionals undertaking them.

#### 4.2.4. Negative Impact of Goal Conflict on QQ

This study found that Goal Conflict exacerbates professionals’ QQ, a finding consistent with related academic studies. Factors that lead to Goal Conflict include too many goals, conflict between roles, and conflict of personal values [66]. Bakker stated that when professionals face role ambiguity and role conflict at work, they are unclear about their responsibilities in the organization [67]. And these can constantly drain professionals’ personal energy and make them feel exhausted [68]. Goal Conflicts may also trigger health outcomes due to decreased job satisfaction, somatic complaints, and anxiety/insomnia [66]. Therefore, organizations can maintain the level of psychological well-being of their professionals and increase their job satisfaction by minimizing goal conflict and paying attention to the unintended consequences that may result from any motivational interventions.

## 5. Conclusions

This study shows that the phenomenon of QQ has become a problem that generally plagues the primary health workforce and requires urgent attention. Second, goal characteristics can be divided into 5 categories. Third, it also confirms the significant effect of goal setting on professionals’ psychological health. Specifically, Goal Specificity and Identity, Goal Fulfillment and Organizational Support can significantly reduce the likelihood of QQ. In contrast, Goal Difficulty, Goal Conflict, and Goal Attainability were found to significantly increase the possibility of QQ. This finding suggests that in practical management settings, the key to effective goal management is to find and maintain a reasonable range for goal setting, by ensuring not only sufficiently specific and moderately difficult goals that are also attainable, but also by securing professional recognition and acceptance and strong organizational support. Such an approach can comprehensively enhance professionals’ intrinsic motivation and lower QQ levels. By systematically linking multidimensional goal characteristics with QQ in the under-researched context of primary healthcare, this study addresses a critical gap in the existing literature, which has predominantly focused on discrete attributes such as goal specificity and difficulty within sports and educational psychology. The present research shifts the perspective from examining isolated attributes to identifying integrated patterns of goal characteristics through the classification of five distinct clusters, thereby providing a richer theoretical framework for understanding the complex mechanisms through which goal setting influences workplace psychology and behavior.

It is particularly important to note that this study was conducted within the unique context of primary healthcare in China, where the system operates under strong government leadership and tightly layered management. This institutional context directly shapes goal-setting processes: primary healthcare institutions set their work goals in accordance with the performance evaluation indicators established by higher-level health departments. Consequently, employees may perceive these pre-defined, explicit goals as non-negotiable job requirements. This top-down process can constrain their sense of autonomy and participation in goal setting, thereby making individual psychological responses—such as goal identity, acceptance, and commitment—critically important. Therefore, while the findings of this study reveal universal relationships between goal setting and QQ, they also provide an important contextualized case for understanding the formation mechanisms of employee behavior under specific institutional and cultural conditions.

This study also has some limitations. First, the cross-sectional design precludes definitive causal inference; while significant associations are identified between goal characteristics and QQ, their causal nature requires verification through longitudinal research. Second, although the adapted scales demonstrated credible reliability and validity in this study, their origin as general instruments lacking systematic validation within Chinese primary healthcare context may affect measurement precision. Certain conceptual nuances or contextual factors specific to this setting may not have been fully captured, potentially influencing the accuracy and interpretability of the measured constructs. Third, the sample for this study was drawn from primary healthcare institutions in China’s poverty alleviation demonstration counties. It remains unclear whether the findings can be generalized to other populations.

In summary, future research should extend the present work along several promising avenues. First, longitudinal designs are essential for clarifying the causal relationships and temporal dynamics between goal characteristics and QQ. Specifically, future studies could employ multi-wave panel surveys or experience-sampling methodologies to test the proposed causal mechanisms. Second, a mixed-methods approach is recommended to explore organizational micro-foundations. For example, in-depth interviews or focus groups could help identify key contextual factors—such as leadership styles, team climate, and communication practices—that shape how goals are perceived and influence employee attitudes. Finally, cross-cultural comparative studies across diverse healthcare systems could test the generalizability of our findings and identify cultural or institutional moderating factors.

## Figures and Tables

**Table 1:** Distribution of goal characteristics and QQ.

Variables	Mean	Variance	S.D.
Goal characteristics			
Goal speciality	4.30	0.34	0.59
Goal difficulty	2.66	0.28	0.53
Goal identity	4.23	0.45	0.67
Goal acceptance	4.09	0.20	0.55
Goal commitment	4.13	0.22	0.47
Goal attainability	3.75	0.28	0.53
Goal conflict	3.18	0.89	0.94
Organization facilitation of goal achievement	4.16	0.34	0.58
Quiet quitting	2.12	0.36	0.60

**Table 2:** Univariate analysis of characteristics on QQ.

Characteristics	Number/Percentage (%)	QQ		t	*p*
		Mean	SD		
Genders				4.58	0.03
Male	189/36.35	2.19	0.65		
Female	331/63.65	2.08	0.56		
Health status				2.55	0.04
Very poor	1/0.2	2.33			
Poor	1/0.2	2.67			
General	106/20.4	2.22	0.64		
Good	221/42.5	2.15	0.56		
Healthy	191/36.7	2.02	0.6		
Job position				2.84	0.02
Clinical Departments	179/34.42	2.22	0.62		
Clinical Inspection Departments	71/13.65	2.15	0.65		
Hospital Administration Departments	78/15.00	2.05	0.61		
Public Health Departments	118/22.69	2.00	0.51		
Other Departments	74/14.23	2.12	0.56		
Length of service				1.82	0.002
0~	249/47.88	2.08	0.59		
10~	107/20.58	2.13	0.66		
20~	91/17.5	2.13	0.55		
30~	70/13.46	2.23	0.58		
40~60	3/0.58	2.03	0.80		
Type of employment				7.39	0.007
On staff	296/56.92	2.18	0.60		
Contract staff	224/43.08	2.04	0.57		

**Table 3:** Distribution of goal categories.

Goal Categories	Mean	Variance	S.D.
Category 1—Goal Specificity and Identity	4.26	0.38	0.61
Category 2—Goal Fulfillment and Organizational Support	4.13	0.22	0.47
Category 3—Goal Difficulty	2.66	0.28	0.53
Category 4—Goal Attainability	3.18	0.89	0.94
Category 5—Goal Conflict	3.75	0.28	0.53

**Table 4:** The correlation between goal categories and QQ.

	(1)	(2)	(3)	(4)	(5)	QQ
(1) Goal Specificity and Identity						
(2) Goal Fulfillment and Organizational Support	0.622 ***					
(3) Goal Difficulty	−0.082	−0.154 ***				
(4) Goal Attainability	0.022	−0.028	0.355 ***			
(5) Goal Conflict	0.336 ***	0.548 ***	−0.312 ***	0.071		
QQ	−0.300 ***	−0.384 ***	0.248 ***	0.358 ***	−0.135 **	

Notes: ** *p* < 0.01, *** *p* < 0.001. (1) = Category 1, (2) = Category 2, (3) = Category 3, (4) = Category 4, (5) = Category 5.

**Table 5:** Multidimensional regression analysis of goal categories on QQ.

Variables	QQ	
	Step 1	Step 2
Category 1—Goal Specificity and Identity	−0.117 *	−0.096 *
Category 2—Goal Fulfillment and Organizational Support	−0.431 ***	−0.466 ***
Category 3—Goal Difficulty	0.124 *	0.112 *
Category 4—Goal Attainability	0.117 *	0.185 ***
Category 5—Goal Conflict	0.193 ***	0.142 **
(Constant)	3.017 ***	3.662 ***
Gender		−0.08
Health Status		−0.064 *
Job position		−0.03 *
Length of service		−0.002
Type of employment		−0.081
R^2^	0.282	0.383
F	41.800 ***	22.336 ***

Notes: The table shows the non-standardized regression coefficients; * *p* < 0.05, ** *p* < 0.01, *** *p* < 0.001.

## Data Availability

The data presented in this study are available on request from the corresponding author. The data are not publicly available because they form a part of an ongoing research project which was funded by the Natural Science Foundation of China (72274066), and the full dataset is scheduled for public release upon the project’s completion in 2027. Prior to that, data can be shared with qualified researchers subject to a reasonable request and necessary agreements.
